# Safety of inter-facility transport strategies for patients referred for severe acute respiratory distress syndrome

**DOI:** 10.1186/s12873-023-00901-y

**Published:** 2023-11-04

**Authors:** Malik Haoutar, David Pinero, Hodane Yonis, Eric Cesareo, Mehdi Mezidi, Olivier Peguet, Karim Tazarourte, Matteo Pozzi, Pierre-Yves Dubien, Jean-Christophe Richard, Laurent Bitker

**Affiliations:** 1 Service de Médecine Intensive – Réanimation, Hôpital de La Croix Rousse, 104, Grande Rue de La Croix Rousse, 69004 Lyon, France; 2 SAMU 69, Hôpital Edouard Herriot, Hospices Civils de Lyon, Lyon, France; 3 Service de Médecine d’Urgence, Hôpital Edouard Herriot, Hospices Civils de Lyon, Lyon, France; 4 INSERM 1290 RESHAPE, Université Claude Bernard Lyon 1, Lyon, France; 5 Service de Chirurgie Cardiaque, Hôpital Louis Pradel, Hospices Civils de Lyon, Lyon, France; 6 Univ Lyon, INSA-Lyon, Université Claude Bernard Lyon 1, CNRS, Inserm, CREATIS UMR 5220, U1294 Villeurbanne, France

**Keywords:** Acute respiratory distress syndrome, Prone position, Extra-corporeal membrane oxygenation, Inter-facility transport, Intensive care

## Abstract

**Background:**

Inter-facility transport of patients with acute respiratory distress syndrome (ARDS) in the prone position (PP) is a high-risk situation, compared to other strategies. We aimed to quantify the prevalence of complications during transport in PP, compared to transports with veno-venous extracorporeal membrane oxygenation (VV-ECMO) or in the supine position (SP).

**Methods:**

We performed a retrospective, single center cohort study in Lyon university hospital, France. We included patients ≥ 16 years with ARDS (Berlin definition) transported to an ARDS referral center between 01/12/2016 and 31/12/2021. We compared patients transported in PP, to those transported in SP without VV-ECMO, and those transported with VV-ECMO (in SP), by a multidisciplinary and specialized medical transport team, including an emergency physician and an intensivist. The primary outcome was the rate of transport-related complications (hypoxemia, hypotension, cardiac arrest, cannula or tube dislodgement) in each study groups, compared using a Fisher test.

**Results:**

One hundred thirty-four patients were enrolled (median PaO_2_/FiO_2_ 70 [58–82] mmHg), of which 11 (8%) were transported in PP, 44 (33%) with VV-ECMO, and 79 (59%) in SP. The most frequent risk factor for ARDS in the PP group was bacterial pneumonitis, and viral pneumonitis in the other 2 groups. Transport-related complications occurred in 36% (*n* = 4) of transports in PP, compared to 39% (*n* = 30) in SP and 14% (*n* = 6) with VV-ECMO, respectively (*p* = 0.33). VV-ECMO implantation after transport was not different between SP and PP patients (*n* = 7, 64% vs. *n* = 31, 39%, *p* = 0.19).

**Conclusions:**

In the context of a specialized multi-disciplinary ARDS transport team, transport-related complication rates were similar between patients transported in PP and SP, while there was a trend of lower rates in patients transported with VV-ECMO.

**Supplementary Information:**

The online version contains supplementary material available at 10.1186/s12873-023-00901-y.

## Background

In moderate to severe acute respiratory distress syndrome (ARDS), prone positioning (PP) has demonstrated a beneficial impact on respiratory mechanics, oxygenation, and survival [1–3]. In the most severe cases of ARDS, veno-venous extracorporeal membrane oxygenation (VV-ECMO) techniques may be indicated but its use remains limited to regional referral centers, which implies transporting patients between facilities [4].

Lyon university hospital (France) has set up a mobile extracorporeal respiratory assistance team, composed of 8 expert physicians (4 emergency physicians, 4 intensivists) working in a bi-disciplinary duo. It has the vocation of offering tele- and on-site expertise as well as secured transport of ARDS patients, be it with or without VV-ECMO. In some patients, the team may ponder to carry out the transport in PP. Indeed, PP may improve oxygenation, but carries the theoretical risk of difficult airway management, accidental extubation, and hemodynamic and respiratory incidents [3, 5, 6]. Yet, the impact of PP on patient security remains unknown when compared to transfer in the supine position with or without VV-ECMO.

We hypothesized that transport in PP without VV-ECMO was not associated with an increased incidence of adverse events during transport if performed by a specialized team, compared to patients transported with VV-ECMO or in the supine position without extracorporeal support. Hence, the primary objective of the study was to report the rate of transport complications based on the transport modality of severe ARDS patients.

## Patients and methods

### Study design

We performed a single center, retrospective cohort study reporting patients transported with ARDS referred to the regional referral intensive care unit (ICU) of the Croix-Rousse hospital (Lyon, France). All study procedures were performed as per French regulatory institutions and guidelines and in accordance with the declaration of Helsinki. The study was approved by the Hospices Civils de Lyon ethics committee (CSE _HCL, IRB 00013204, reference number 22_796) prior to data collection. Informed consent was obtained by requesting the non-opposition of participants to the retrospective use of their data, systematically sought by way of an information letter detailing the study protocol sent to them and/or their next-of-kin. The report was written following the STROBE checklist [7].

### Study population

We considered eligible for inclusion all patients aged 16 or more with confirmed ARDS and who were referred and transported to the ARDS referral center between 01/12/2016 and 31/12/2021. ARDS was defined using the Berlin definition [8]. The inclusion period included the first 2 years of the COVID-19 pandemic in France (start date March 17^th^, 2020). Exclusion criteria were previous inclusion in the same study during a prior ICU stay, and lack of consent for data utilization by patients or their legal representative.

### Transport of referred ARDS patients

Referral was requested by the addressing ICU when the first lines of therapies were deemed unsuccessful by the treating ICU physician, and that he or she deemed that the patient required expert care by a tertiary center and/or extracorporeal respiratory support. Referral was assessed by a mixed team of emergency and ICU physicians with experience and expertise in ARDS management and transport of critically ill patients with ARDS (the ECMO team). After application of a dedicated stepwise respiratory management protocol (described below), the team could either transport the patient in SP without VV-ECMO, in SP with VV-ECMO, or in PP without VV-ECMO (corresponding to the 3 study groups: SP, VV-ECMO, and PP). Transport was performed by land, using mobile ICU dedicated ambulances.

### Study primary and secondary outcomes

The primary outcome was the prevalence of transport-related complications in the 3 study groups. The transport period was defined as the time between the arrival of the transport team at the addressing ICU to the time of arrival to the referral ICU. Hence, transport complications may encompass any event occurring during the pre-transport optimization period. We defined transport-related complications as any life-threatening deterioration in the clinical status of the patient: hypoxemia, hypotension, hemorrhage, accidental removal of endotracheal tubes, catheters or VV-ECMO cannulas, and cardiac arrest. Any death occurring after transport (same calendar day) was a posteriori identified as being a transport-related complication or not, after review by 2 investigators (LB and JCR). Hypoxemia was defined as any episode of percutaneous oxygen saturation [S_p_O_2_] < 88% for more than 10 min; hypotension as any episode of mean arterial pressure < 65 mmHg for more than 10 min. We also considered the occurrence of facial pressure soar.

The secondary study endpoints included respiratory and hemodynamic status and management before, during and after transport (see below for study time points definition after transport), organ failure severity evaluated by the SOFA score after transport, ventilator-free days (VFD) at day 60, the rate of VV-ECMO cannulation after transport in patients transported without VV-ECMO, and ICU and hospital mortality [9, 10].

### Management of patients in SP without VV-ECMO prior to and during transport

All transports were performed using a Monnal T60 ventilator (Air Liquide, Paris, France), with a heat and moisture exchanger. Patients were not disconnected from the ventilator at any time during transport. If disconnection from the ventilator was mandated, the tracheal tube was clamped at end-expiration to avoid alveolar collapse. Patients’ ventilatory settings prior to transport was protocolized, the details of which are given in Supplemental Methods 1. In case of persistent hypoxemia (S_p_O_2_ < 88%) despite initial ventilatory optimization, a protocolized procedure aiming to optimize PEEP and V_T_ to achieve a S_p_O_2_ ≥ 88% was applied (Supplemental Fig. 1). If the patient had a S_p_O_2_ < 88% despite optimization, the decision was made to either transport the patient in PP or with VV-ECMO.

### Eligibility and management of patients with VV-ECMO

VV-ECMO cannulation could either be performed by the expert team at the addressing ICU (“on- site”), or after patient’s transport to the referral ICU. In case the decision was made to cannulate prior to transport, the ECMO team (i.e. an intensivist and an emergency physician) was transported to the addressing ICU for on-site cannulation by way of a percutaneous approach. Clinical criteria for VV-ECMO were a P_a_O_2_/F_i_O_2_ (oxygen arterial partial pressure to O_2_ inspired fraction ratio) < 50 mmHg for > 3 h (severe refractory hypoxemia), a P_a_O_2_/F_i_O_2_ < 80 mmHg for > 6 h (refractory hypoxemia) or an arterial pH < 7.15 for > 6 h with PaCO_2_ (arterial carbon dioxide partial pressure) > 60 mm Hg and respiratory rate increased to 35 min^−1^, resulting from mechanical ventilation settings adjusted to keep plateau pressure ≤ 30 cm H_2_O (refractory hypercapnia) [4]. These criteria were the same used to cannulate patients already admitted to the referral center. Of note, a PP trial was systematically suggested by the referral center to be performed on-site prior to sending the ECMO team.

Patients were not eligible for VV-ECMO if therapeutic limitation decisions were already in place, if they had left ventricular ejection fraction < 25%, or in case of a chronic disease with < 5-year survival. Patients were not eligible in case of uncontrolled shock with increasing lactate concentrations despite hemodynamic optimization, or if their SOFA score was > 18. Decision of implantation was discussed in case of age > 75 years, of chronic respiratory disease, or if the duration of mechanical ventilation was > 7 days. In the context of the COVID-19 pandemic, the French health regulation authority also released recommendations to spare VV-ECMO resources (based on age and duration of invasive ventilation at the time of VV-ECMO eligibility assessment).

The final decision on the modality of transport (with or without VV-ECMO) was made by the ECMO team, based on the criteria described above, and on their expert decision to delay VV-ECMO implantation to after transport to the referral center especially in patients eligible to VV-ECMO without identified contra-indications. VV-ECMO membranes, pumps and technique of cannulation are described in Supplemental Methods 2, along with mechanical ventilation settings during VV-ECMO.

### Transport in the prone position

If the patient had a S_p_O_2_ < 88% in SP despite ventilatory optimization and a S_p_O_2_ > 90% in PP, the ECMO team evaluated the feasibility of transport in PP without implementing VV-ECMO. Contra-indications to transport in PP were unstable cervical fracture, and obesity > 35 kg.m^−2^. In practice, the patient was prone positioned in the ICU bed; a stabilization period of 15 min was then applied, at the end of which the team concluded on the response to PP based on S_p_O_2_ (> 88%) or P_a_O_2_ (> 55 mmHg) targets. The patient was then transferred in PP on the on the mobile ICU stretcher, with continuous firm support of the endotracheal tube.

### Post-transport patient management

Patients’ management after transport in the referral ICU followed the French and international ARDS recommendations, and is described in terms of ventilatory and VV-ECMO management (settings, weaning) in Supplemental Methods 3 [11, 12].

### Data collection

Day-0 corresponded to the day of transport. Variables corresponding to pre-transport measurements were those measured on transport day, before the patient’s departure from the addressing ICU. Day 1, day 3 and day 7 corresponded to the first, third and seventh calendar day in the referral ARDS center. Supplemental Methods 4 further describe data collection strategy.

### Statistics

A convenience sample of all transported patients between the date of first transport by the ECMO team and the date of study declaration to regulatory authorities was considered. A *p*-value < 0.05 was considered for statistical significance. All analysis were performed using the R software (R foundation for statical computing) [13]. Continuous data were reported with median and interquartile range, and categorical data with count and percentage. The observed rate of the primary outcome is accompanied with its 95% confidence interval in the 3 study groups, determined using the Wilson method. Missing values are reported in Supplemental Table 1.

Variables were compared between the 3 study groups using a Kruskall-Wallis test for continuous variables, and a Fisher test for categorical variables. A post hoc analysis with pairwise comparison (Dunnett test for continuous variables or a logistic regression model for categorical variables, using in both cases the PP group as the reference group) was performed if the *p*-value of the Kruskall-Wallis or the Fisher test was < 0.20.

Variables repeatedly measured over time were compared between groups using mixed effects linear regression, with the interaction of time and study group as fixed effects, and the patient’s identification number as a random intercept. In case of a significant interaction, a post hoc pairwise comparison between study groups was performed at each time points, using the PP group as the reference (Bonferroni’s method).

Risk factors of transport-related complications were identified using a multivariate logistic regression of variables associated with complications. The selection of variables inserted in the model was based on their clinical relevance. The multivariate model was determined using a backward stepwise strategy, selecting variables that significantly improved the model. The distance in kilometers from the referral ICU was included as an offset into the multivariate model. Multicollinearity was identified if a variance inflation factor was > 3 and the variable subsequently excluded. Interactions were systematically checked for in the multivariate analysis. *p* values were determine using bootstrapping with 500 samples.

## Results

### Study population

Between 01/12/2016 and 31/12/2022, 136 patients were transferred to the regional ARDS referral center (Supplemental Fig. 2). Two patients were excluded due to uncertainty of their body position during transport, leaving 134 participating patients (93 (69%) males, median age 55 [44–64] years) (Table 1), with a lowest P_a_O_2_/F_i_O_2_ ratio on transport day of 67 [56–75] mmHg (Table 2). Most patients presented with severe ARDS (*n* = 127, 95%), with the most frequent ARDS risk factor being viral pneumonia (*n* = 106, 79%, Supplemental Table 2). The delay between the date of admission in the addressing ICU admission and transport was 5 [2-8] days (Table 3). Supplemental Table 3 reports the theoretical VV-ECMO indications and contra-indications in patients transported without VV-ECMO.

**Table 1:** Population’s characteristics

	Whole population	Prone position	ECMO	Supine position	*p*
	*n* = 134	*n* = 11	*n* = 44	*n* = 79	
Age, years	55 [44–64]	63 [48–68]	52 [45–60]	56 [44–64]	0.46
Gender male, n (%)	93 (69%)	6 (55%)	31 (70%)	56 (71%)	0.52
Weight, kg	89 [78–105]	77 [64–83]*^,†^	90 [82–104]	90 [78–105]	0.03
Height, cm	172 [161–177]	164 [160–170]*^,†^	172 [164–177]	172 [161–177]	0.13
BMI, kg.m^−2^	31 [27–35]	26 [22–34]	31 [27–35]	31 [27–35]	0.23
Predicted body weight, kg	66 [56–72]	57 [53–66]*^,†^	67 [57–72]	67 [56–72]	0.17
SAPS II score (addressing ICU)	58 [50–64]	62 [44–71]	59 [53–64]	57 [50–64]	0.64
Charlson score	2 [0–3]	2 [1-3]	1 [0–3]	2 [1-3]	0.41
**Comorbidities**					
Diabetes, n (%)	37 (28%)	2 (18%)	11 (25%)	24 (30%)	0.70
NYHA III-IV heart failure, n (%)	3 (2%)	0 (0%)	1 (2%)	2 (3%)	0.99
Chronic pulmonary disease*, n (%)	29 (22%)	0 (0%)	8 (18%)	21 (27%)	0.10
Asthma, n (%)	9 (7%)	0 (0%)	2 (5%)	7 (9%)	0.56
COPD, n (%)	8 (6%)	0 (0%)	0 (0%)	8 (10%)	0.07
Cystic fibrosis, n (%)	1 (1%)	0 (0%)	0 (0%)	1 (1%)	0.99
Stage V chronic kidney disease, n (%)	2 (1%)	0 (0%)	1 (2%)	1 (1%)	0.99
Immunodepression, n (%)	18 (13%)	2 (18%)	6 (14%)	10 (13%)	0.79
**Admission category (addressing ICU)**					
Medical, n (%)	124 (93%)	11 (100%)	41 (93%)	72 (91%)	0.89
Scheduled surgery, n (%)	4 (3%)	0 (0%)	1 (2%)	3 (4%)	0.99
Emergent surgery, n (%)	4 (3%)	0 (0%)	1 (2%)	3 (4%)	0.99
Trauma, n (%)	3 (2%)	0 (0%)	0 (0%)	3 (4%)	0.65

Data is shown as median [interquartile range] or count (percentage). Missing values were not imputed. Percentage are reported to the whole number of observations of the column, including missing values. *p*-values examine the difference between groups, using a Kruskall and Wallis test (continuous variables) or a Fisher test (categorical variable). A post hoc analysis with pairwise comparison (Dunnett test for continuous variables or a logistic regression model for categorical variables) was performed if the *p*-value of the KW or the Fisher test was < 0.20. *: *p* < 0.05 in post hoc analysis between the PP group and the ECMO group; †:* p* < 0.05 in post hoc analysis between the PP group and the SP group. BMI: body mass index; VV-ECMO: veno-venous extracorporeal membrane oxygenation; NYHA: New York Heart Association dyspnea scale; PP: prone position; SAPS: simplified acute physiology score; SP: supine position

**Table 2:** Patients’ characteristics on transport day (prior to transport)

	Whole population	Prone position	ECMO	Supine position	*p*
	*n* = 134	*n* = 11	*n* = 44	*n* = 79	
**Arterial blood gas on transport day (before transport)**					
S_p_O_2_ closest to transport, %	92 [89–94]	92 [87–94]	91 [88–93]	93 [91–95]	0.01
pH closest to transport	7.34 [7.26–7.40]	7.28 [7.22–7.40]	7.34 [7.26–7.40]	7.35 [7.28–7.40]	0.56
P_a_O_2_/F_i_O_2_ closest to transport, mmHg	70 [58–82]	69 [65–73]	58 [52–73]	74 [67–85]	< 0.01
Lowest P_a_O_2_/F_i_O_2_ on transport day, mmHg	67 [56–75]	68 [60–73]*	57 [50–67]	70 [62–80]	< 0.01
Lowest P_a_O_2_/F_i_O_2_ in SP on transport day, mmHg	75 [64–90]	72 [61–74]	72 [60–84]	80 [67–96]	0.12
Lowest P_a_O_2_/F_i_O_2_ in PP on transport day, mmHg	82 [72–104]	85 [74–97]	76 [64–94]	84 [77–107]	0.10
P_a_O_2_/F_i_O_2_ response to PP, n (%)	33 (25%)	3 (27%)	13 (30%)	17 (22%)	0.492
P_a_CO_2_ closest to transport, mmHg	52 [45–62]	50 [46–58]	55 [48–67]	51 [45–59]	0.20
**Ventilatory settings on transport day (parameters before transport and closest to it)**					
Tidal volume, ml.kg^−1^ PBW	6 [4.9–6.4]	6.5 [6.4–7.6]*,†	5.4 [4–6.3]	6.0 [5.1–6.4]	< 0.01
Set PEEP, cmH2O	12 [10–14]	14 [12–15]	12 [8–15]	12 [10–14]	0.58
Plateau pressure, cmH_2_O	28 [24–30]	26 [20–30]	26 [22–32]	28 [26–30]	0.34
Driving pressure, cmH_2_O	15 [12–18]	14 [9–16]	15 [11–18]	16 [12–20]	0.38
**Patient management up to transport day (received at any time before transport)**					
Neuromuscular blockade, n (%)	134 (100%)	11 (100%)	44 (100%)	79 (100%)	-
Inhaled nitric oxyde, n (%)	87 (65%)	9 (82%)	30 (68%)	48 (61%)	0.39
Prone position, n (%)	122 (91%)	10 (91%)	43 (98%)	69 (87%)	0.24
Number of prone sessions before transport day	2 [1–3]	2 [1–3]	2 [1–3]	2 [1–3]	0.87
Oxygenation response to PP	33 (25%)	3 (27%)	13 (30%)	17 (22%)	0.49
**Severity of disease on transport day (worst value before transport)**					
Vasopressors, n (%)	69 (51%)	5 (45%)	20 (45%)	44 (56%)	0.49
Renal replacement therapy, n (%)	8 (6%)	0 (0%)	2 (5%)	6 (8%)	0.86
SOFA score on transport day, n (%)	11 [8–13]	11 [9–15]	9 [8–13]	11 [8–12]	0.37
Arterial lactate, mmol.L^−1^	1.6 [1.3–2.3]	2.1 [1.6–2.7]	1.6 [1.3–2.5]	1.6 [1.3–2]	0.29

Data is shown as median [interquartile range] or count (percentage). Missing values were not imputed. Percentage are reported to the whole number of observations of the column, including missing values. *p*-values examine the difference between groups, using a Kruskall and Wallis test (continuous variables) or a Fisher test (categorical variable). A post hoc analysis with pairwise comparison (Dunnett test for continuous variables or a logistic regression model for categorical variables) was performed if the *p*-value of the KW or the Fisher test was < 0.20. *: *p* < 0.05 in post hoc analysis between the PP group and the ECMO group; †:* p* < 0.05 in post hoc analysis between the PP group and the SP group. *VV-ECMO* veno-venous extracorporeal membrane oxygenation, *F*_*i*_*O*_*2*_ O_2_ inspired fraction, *ICU* intensive care unit, *P*_*a*_*O*_*2*_ arterial partial pressure in O_2_, *P*_*a*_*CO*_*2*_ arterial partial pressure in CO_2_, *PEEP* positive end-expiratory pressure, *PP* prone position, *SP* supine position, *SOFA* sepsis-related organ failure assessment score, *S*_*p*_*O*_*2*_ O_2_ percutaneous saturation

**Table 3:** Transport description

	Whole population	Prone position	VV-ECMO	Supine position	*p*
	*n* = 134	*n* = 11	*n* = 44	*n* = 79	
Delay between ICU admission and transport day, days	5 [2–8]	3 [2–7]	4 [1–8]	5 [2–9]	0.73
Delay between intubation and transport day, days	2 [1–5]	3 [0–4]	2 [0–5]	2 [1–5]	0.83
Transported during the COVID-19 pandemic	100 (75%)	3 (27%)*,†	36 (82%)	61 (77%)	< 0.01
Transported at night or non-working days, n (%)	71 (53%)	7 (64%)	22 (16%)	42 (53%)	0.74
Distance, km	44 [7–89]	46 [10–82]	46 [7–107]	44 [7–89]	0.21
< 10 km, n (%)	39 (29%)	1 (9%)	12 (27%)	26 (33%)	0.26
> 100 km, n (%)	25 (19%)	2 (18%)	13 (30%)	10 (13%)	0.07
Duration, min	45 [30–62]	40 [27–58]	60 [41–70]	40 [30–60]	0.09
**Transport supervision**					
ARDS centre expert, n (%)	40 (30%)	3 (27%)*,†	35 (80%)	2 (3%)	< 0.01
Mobile ICU expert, n (%)	40 (30%)	4 (36%)	20 (45%)	16 (20%)	0.01
Secondary reinforcement by expert, n (%)	6 (4%)	1 (9%)	2 (5%)	3 (4%)	0.51
Transported with inhaled NO, n (%)	57 (43%)	10 (91%)*	3 (7%)	44 (56%)	< 0.01
**Respiratory parameters and ventilatory settings during transport**					
S_p_O_2_, %	92 [88–95]	94 [91–96]	93 [90–95]	90 [87–95]	0.34
Estimated P_a_O_2_/F_i_O_2_^§^	72 [58–86]	77 [64–86]	76 [62–92]	69 [53–86]	0.36
F_i_O_2_ or F_m_O_2_, %	100 [88–100]	100 [92–100]	100 [82–100]	100 [88–100]	0.96
Tidal volume, ml.kg^−1^ PBW	5.4 [4.1–6.1]	5.9 [5.5–6.4]*	1.3 [1–2.1]	5.7 [4.8–6.2]	< 0.01
Set PEEP, cmH_2_O	14 [10–16]	15 [12–16]	13 [10–15]	13 [10–16]	0.38
Plateau pressure, cmH_2_O	27 [22–30]	30 [26–34]*	21 [20–22]	28 [26–31]	0.01
Driving pressure, cmH_2_O	14 [10–18]	14 [14–16]	6 [4–12]	16 [14–18]	0.01
**Respiratory interventions during transport**					
F_i_O_2_ change, n (%)	22 (16%)	1 (9%)	6 (14%)	15 (19%)	0.71
Tidal volume change, n (%)	31 (23%)	3 (27%)	9 (20%)	19 (24%)	0.86
PEEP change, n (%)	37 (28%)	3 (27%)	10 (23%)	24 (30%)	0.63
Inhaled NO dose change, n (%)	18 (13%)	0 (0%)	4 (9%)	14 (18%)	0.19
Prone to supine positioning during transport, n (%)	1 (1%)	0 (0%)	0 (0%)	1 (1%)^#^	0.99
Supine to prone positioning during transport, n (%)	1 (1%)	0 (0%)	0 (0%)	1 (1%)	0.99

Data is shown as median [interquartile range] or count (percentage). Missing values were not imputed. Percentage are reported to the whole number of observations of the column, including missing values. *p* values examine the difference between groups, using a Kruskall and Wallis test (continuous variables) or a Fisher test (categorical variable). A post-hoc analysis with pairwise comparison (Dunnett test for continuous variables or a logistic regression model for categorical variables) was performed if the p value of the KW or the Fisher test was < 0.20. §: P_a_O_2_/F_i_O_2_ ratio estimated from the lowest measured S_p_O_2_ during transport; *: *p* < 0.05 in post hoc analysis between the PP group and the VV-ECMO group; †:* p* < 0.05 in post hoc analysis between the PP group and the SP group. #: one patient had a failed PP attempt with a hypoxemia episode (as part of the optimization strategy before transport), and was transported in the supine position. *ARDS* acute respiratiry distress syndrome, *VV-ECMO* veno-venous extracorporeal membrane oxygenation, *F*_*i*_*O*_*2*_ oxygen inspired fraction, *F*_*m*_*O*_*2*_ sweep gas O_2_ fraction, *ICU* intensive care unit, *NO* nitric-oxide, *P*_*a*_*O*_*2*_ arterial partial pressure in O_2_, *PEEP* positive end-expiratory pressure, *PP* prone position, *SP* supine position, *S*_*p*_*O*_*2*_ O_2_ percutaneous saturation

### Transport in the prone position

**Fig. 1:**
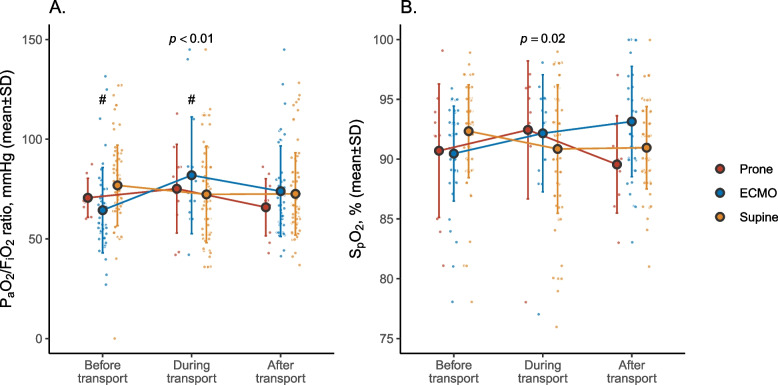
Oxygen response over the transport period. The figure shows the individual measurement (light dots) and mean values of the P_a_O_2_/F_i_O_2_ ratio (panel **A**), and S_p_O_2_ values (panel **B**), in the 3 study groups (prone in red, ECMO in blue, and supine in yellow), before, during and immediately after transportation. In case several values were available at a given time, the most pejorative value is represented. The *p*-value examines the association of the Group by Time interaction term with the parameter of interest, using a mixed effects regression model in which the patient identification number served as random effect. The hashtag (#) indicates a significant difference (*p* < 0.05) between the prone and VV-ECMO group at a given time, in the post hoc pairwise analysis. Also, no difference in P_a_O_2_/F_i_O_2_ or in S_p_O_2_ was observed in the PP group between the different time points. In panel B, no difference between study groups was observed in the post hoc analysis despite the significant interaction. F_i_O_2_: inspired fraction in oxygen; P_a_O_2_: arterial partial pressure in oxygen; S_p_O_2_: percutaneous saturation in oxygen

Rate of transport in the prone position was 8% (*n* = 11) in the cohort. All PP patients had severe ARDS (Supplemental Table 2). Compared to patients transported with VV-ECMO (*n* = 44, 33%) or in SP (*n* = 79, 64%), PP patients has a significantly lower body weight and height (Table 1). There was no difference between groups in the rate of oxygenation response to PP prior to transport (Table 2). The P_a_O_2_/F_i_O_2_ ratio significantly increased between pre-transport and the per-transport period in the VV-ECMO group, while it remained stable in PP patients (Fig. 1). Table 3 describes patients’ clinical status and management during transport.

### Transport complications

**Fig. 2:**
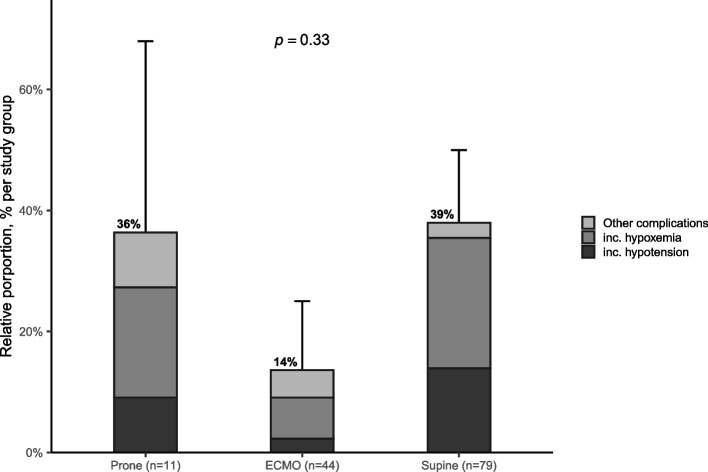
Transport-related complications in each study group. The figure shows the relative proportion, relative to each study group size, of all transport-related complications (height of bar, with percentage in bold) with its 95% confidence interval. Within each bar plot is represented the proportion of hypoxemia (in grey), hypotension (in black) and other complications (light grey). The *p*-value examines the difference between complication frequency between study groups. VV-ECMO: veno-venous extracorporeal membrane oxygenation

Frequency of per-transport complications was 36% (95% confidence interval [12–68]) in the PP group, with no significant difference between study groups (Fig. 2). Principal complications included hypoxemia and hypotension, with 1 reported death related to transport complications (Table 4). A higher cardiovascular SOFA score was significantly associated with an increased risk of per-transport complications (Table 5), while the distance between the addressing center and the referral center was not (Supplemental Fig. 3). The frequency of ventilatory settings change during transport was also similar between groups (Table 3). Patients with COVID-19 demonstrated lesser clinical severity, were more frequently transported with VV-ECMO, and less per-transport complications, compared to non-COVID-19 patients (Supplemental Table 4).

**Table 4:** Complications during transport

	Whole population	Prone position	VV-ECMO	Supine position	*p*
	*n* = 134	*n* = 11	*n* = 44	*n* = 79	
Any complications during transport, n (%)	39 (29%)	4 (36%)	6 (14%)	30 (38%)	0.33
Hypoxemia episode, n (%)	22 (16%)	2 (18%)	3 (7%)	17 (22%)	0.44
Hypotensive episode, n (%)	13 (10%)	1 (9%)	1 (2%)	11 (14%)	0.32
Lowest MAP, mmHg	75 [66–85]	80 [67–83]	80 [68–91]	74 [66–83]	0.49
Unscheduled extubation, n (%)	0 (0%)	0 (0%)	0 (0%)	0 (0%)	-
Unscheduled catheter or VV-ECMO canula desinsertion, n (%)	0 (0%)	0 (0%)	0 (0%)	0 (0%)	-
Cardiac arrest during transport, n (%)	1 (1%)	0 (0%)	1 (2%)	0 (0%)	0.33
Cardiac arrest on calendar day of transport (after transport), n (%)	3 (2%)	0 (0%)	1 (2%)	2 (3%)	0.99
Death on calendar day of transport (after transport), n (%)	5 (4%)	1 (9%)	2 (5%)	2 (3%)	0.44
Death on calendar day of transport related to transport complications, n (%)	0 (0%)	0 (0%)	0 (0%)	1 (1%)	0.99
Facial pressure soars, n (%)	1 (1%)	0 (0%)	0 (0%)	1 (1%)	0.99

Data is shown as median [interquartile range] or count (percentage). Missing values were not imputed. Percentage are reported to the whole number of observations of the column, including missing values. *p*-values examines the difference between groups, using a Kruskall and Wallis test (continuous variables) or a Fisher test (categorical variable). A post hoc analysis with pairwise comparison (Dunnett test for continuous variables or a logistic regression model for categorical variables) was performed if the *p*-value of the KW or the Fisher test was < 0.20. *: *p* < 0.05 in post hoc analysis between the PP group and the ECMO group; †:* p* < 0.05 in post hoc analysis between the PP group and the SP group. VV-ECMO: veno-venous extracorporeal membrane oxygenation; MAP: Mean arterial pressure

**Table 5:** Association of pre-transport variables with the probability of any complications occurring during transport

	**Univariate analysis**		**Multivariate analysis**	
	**OR [95% CI]**	***p***	**OR [95% CI]**	***p***
Age, per 10 year increase	0.86 [0.65–1.15]	0.31	-	-
Study group		0.11		0.14
Prone position	1		1	
VV-ECMO	0.38 [0.07–1.92]		0.27 [0.03–2.33]	
Supine position	1.13 [0.29–4.73]		1.19 [0.21–7.66]	
Cardiovascular SOFA, per 1 point increase	1.39 [1.10–1.77]	< 0.01	1.81 [1.27–2.64]	< 0.01
P_a_O_2_/F_i_O_2_, per 10 mmHg increase	1.04 [0.81–1.32]	0.76	1.20 [0.87–1.68]	0.17
Arterial lactate, per 1 mmol/L increase	1.25 [0.99–1.68]	0.09	1.31 [1.00–1.78]	0.06

The table shows the result of the univariate and multivariate analysis of variables associated with the risk of any complications occurring during transport (*n* = 40/134). No multicollinearity and no interaction were identified in the final multivariate model. Multivariate model C-stat = 0.78 [95% CI: 0.68–0.87]. *95% CI* 95% confidence interval, *F*_*i*_*O*_*2*_ inspired O_2_ fraction, *ICU* intensive care unit, *OR* odds ratio, *P*_*a*_*O*_*2*_ arterial O_2_ partial pressure, *SOFA* sepsis-related organ failure assessment

### Post-transport outcomes

After transport, there was no difference in PP rate of use between study groups (Supplemental Fig. 4), and 7 patients (64%) of the PP group required VV-ECMO, with a median delay after transport of 6 [4-17] hours (Table 6). Four patients in the PP group were alive and free from mechanical ventilation at day-60 and VFD at day-60 were 0 [0–50] days in the PP group, not statistically different from the other 2 groups.

**Table 6:** Clinical outcomes

	Whole population	Prone position	ECMO	Supine position	*p*
	*n* = 134	*n* = 11	*n* = 44	*n* = 79	
ICU length of stay, days	24 [10–38]	16 [8–28]	25 [10–38]	24 [10–40]	0.46
Hospital length of stay, days	30 [16–45]	22 [16–36]	28 [12–42]	35 [18–47]	0.30
ICU death, n (%)	79 (59%)	6 (55%)	29 (66%)	44 (56%)	0.44
In-hospital death, n (%)	80 (60%)	7 (64%)	29 (66%)	44 (56%)	0.50
Death at day-60, n (%)	77 (57%)	7 (64%)	29 (66%)	41 (52%)	0.29
Ventilator-free days at day-60, days	0 [0–26]	0 [0–50]	0 [0–6]	0 [0–34]	0.29
Number alive and free from mechanical ventilation at day-60, n (%)	42 (31%)	4 (36%)	11 (25%)	27 (34%)	0.48
Post-transport ECMO cannulation, n (%)	38 (28%)	7 (64%)^a,b^	-	31 (39%)	0.19
Time to ECMO cannulation, hours	4 [-1–21]	6 [4–17]*	-2 [-3 – -1]	21 [11–80]	< 0.01
Number alive and free from ECMO at day-60, days	22 (16%)	1 (9%)	13 (30%)	8 (10%)	0.25

Data is shown as median [interquartile range] or count (percentage). Missing values were not imputed. Percentage are reported to the whole number of observations of the column, including missing values. *p*-values examines the difference between groups, using a Kruskall and Wallis test (continuous variables) or a Fisher test (categorical variable). For the specific comparison of post-transport ECMO cannulation, we used a Fisher test to compare the rate observed in the PP group and the SP group. A post hoc analysis with pairwise comparison (Dunnett test for continuous variables or a logistic regression model for categorical variables) was performed if the *p*-value of the KW or the Fisher test was < 0.20. a: The indication of post-transport cannulation in the PP group were hypoxia (*n* = 6/11) and hypercapnia (*n* = 1/11). b: 3 of the 4 patients that were never cannulated after transport in the PP group were discharged alive from hospital. *: *p* < 0.05 in post hoc analysis between the PP group and the ECMO group; †:* p* < 0.05 in post hoc analysis between the PP group and the SP group. *VV-ECMO* veno-venous extracorporeal membrane oxygenation, *ICU* intensive care unit, *PP* prone position, *SP* supine position

## Discussion

Over a period of 5 years, 134 ARDS patients were transported to an ARDS referral center, among which 11 were transported in PP. Our results show that 1/ transport-related complications occurred in more than a third of patients transported in PP and in SP, although the first were more severely ill that the latter at time of transport; 2/ patients transported in PP had a high probability of being treated with VV-ECMO after transfer; and 3/ transport with ECMO appeared to reduce the rate of transport-related complications, compared to other modalities.

Our study shows that the decision to transport in PP was unfrequently made. No study to date has evaluated the safety or efficacy of protocolized transport decision-making in ARDS patients. However, our results show that, based on clinical judgment, these patients benefited from relatively safe transport to the referral center, where they were able to receive tertiary level treatments such as VV-ECMO. Our results show that the factors influencing decision making were morphometric on the one side (lower weight) but also logistic. Indeed, patients in the ECMO group were more frequently transported during daytime on weekdays compared to PP patients, and over slightly longer distances [14]. It is also possible that patients transported in PP were those with borderline ECMO indications which would have potentially benefited from revaluation after further expertise by the referral center. Finally, our results show that the COVID-19 pandemic has led to a drastic change in patient management and decision making, due to a large increase in patient volume and the team’s experience. This explains the imbalance between viral and non-viral ARDS risk factors between study groups.

Even though previously published data report a relatively low incidence of complications, patient safety is a major concern when considering inter-facility transport of patients with ARDS. The incidence of complications in PP-transported patients ranges from 14 to 56% of transports in the literature (i.e. encompassing the observed rate in our study), with the main mechanisms being hemodynamic or respiratory complications [5, 6, 15]. None reported accidental tube or cannula dislodgment or per-transport cardiac arrest. In a large cohort of 431 ARDS patients transported in SP with or without ECMO (but none in PP) with a slightly lower clinical severity compared to ours, less than 12% presented with a critical transport-related event, the majority of which were hypoxemia or hypotensive episodes, and 2 accidental extubations [16]. Unfortunately, these important studies lacked a control group to allow comparison. We also demonstrated that the risk of complications was independently associated with pre-transport hemodynamic impairment, but not with the transport strategy, in the multivariate analysis. Finally, comparability of transport conditions is however hampered by the fact that most critical care transport (CCT) societies in Northern America are manned by nurses and trained paramedics and are frequently airborne, while most transports are medicalized in Europe and use land ambulances [5, 6, 14–16]. A direct consequence of medicalized transport is the higher rate of per-transport ventilatory modifications in our cohort, compared to the Boston cohort, but with little differences in severe adverse events compared to non-medical supervision of transport [16].

We also observed that patients transported in PP were frequently treated with VV-ECMO once admitted to the referral ICU. Only a third of all transported patients in the Boston cohort were treated with VV-ECMO after transport, which is nearly half the number we report; this could be related to slightly less severely hypoxemic patients in this cohort as compared to ours [5]. Of note, 3 of the 4 patients that were not cannulated in the PP group after transport were discharged alive, demonstrating the potential relevance of this conservative strategy.

Compared to the existing literature, the high mortality rate observed in our cohort go with the fact that our selected patients were more severely ill prior to transport, and the potential requirement for VV-ECMO, as suggested by the reported P_a_O_2_/F_i_O_2_ ratios compared to other studies [1, 5, 6, 15, 17]. Also, patients’ outcome in the 3 study groups were similar in terms of ventilation duration and survival, suggesting the absence of negative impact of this transport strategy, keeping in mind that ARDS risk factors significantly differed between study groups. Another hypothesis was that patients’ outcome may have been affected by the delay prior to referral to Lyon tertiary center. However, the median delay between intubation and transfer was 2 days in our work, significantly lower than those reported previously [5, 16].

COVID-19 ARDS was the principal cause of ARDS in our study, a subtype of ARDS known to be associated with worse outcome [1, 17]. COVID-19 patients in our cohort had lower SOFA scores at time of transport, compared to non-COVID-19 patients, and presented less frequently with per-transport complications. We hypothesized that this is related to the increase in patient volume related to the pandemic which led to: 1-better triage and selection of referred patients due to resource and logistic constraints; 2- increasing clinician expertise in the per-transport management; and 3- lesser clinical severity of COVID-19 patients as compared to non-COVID-19 patients. Finally, conclusions regarding the specific consequences of COVID-19 ARDS transfer strategy are hampered by the low numbers of COVID-19 patients transported in PP during the pandemic in our study.

Our results suggest that inter-facility transfers performed by an expert team of trained nurses and physicians allow the relatively safe inter-hospital transport in PP of patients with severe ARDS, compared to transport in SP. Although not statistically significant, the lower rate of complications in the VV-ECMO group does suggest its beneficial effect on transport safety on this very severe category of transported patients. On the other hand, a third of patients transported in PP did not require VV-ECMO after transport and would have been otherwise implanted before transport if solely indicated to secure the transfer. Yet, VV-ECMO is associated with longer ICU length of stay and mechanical ventilation duration, and its benefits in terms of transport complication prevention must be weighted by clinicians against the potential morbidity of this extracorporeal technique. Also, PP transport is likely an option if ECMO teams or consumables are scarce, or if ECMO indications are not certain and require further expertise at the referral center. These elements mandate the prospective evaluation of strategies to identify patients eligible to be transported in PP using dedicated protocols assigning patients to a transport modality based on predefined criteria, and evaluating both per-transport complications but also patients’ outcomes after transport [5]. Especially, hemodynamic stability and circulatory impairment at time of referral should guide clinicians in deciding the best transfer strategy, to decrease the rate of per-transport complications in this population. Finally, our study also suggests that the COVID-19 pandemic induced a change in practice regarding transport modalities, due to improvements in logistics and organization, allowing early on-site VV-ECMO cannulation, despite an increased burden of human and material resources. Finally,

This study has several strengths. First, our work is the only to report 2 control groups, as well as detailed and longitudinal respiratory mechanics and ventilatory management of transported patients. Second, ventilatory management prior to and during transport was protocolized allowing comparison between groups and across time. Third, the criteria for complications during transfer were extrinsically recognized for their relevance in previous publications [5, 6].

This study also has several limitations. First, this study was a single center study and the conclusions drawn from our cohort may not be generalizable to other centers. Also, the sample size related to the use of a convenience sample may have been insufficient to detect a significant difference in transport-related complications due to insufficient statistical power. Second, this study was retrospective by design. However, protocolized management on the one hand, and a predefined data collection plan on the other, would have limited selection or confusion biases. Third, criteria and strategies of transport in PP were not protocolized and were left to the discretion of the ECMO team, after application of the optimization algorithm in SP and evaluation of VV-ECMO eligibility. This may have led to a potential selection bias, and subsequently affected the reported rate of per-transport complications. Finally, the data collection was carried out over a relatively long period (5 years) with potential changes in practice and techniques, which may have led to a temporal bias. However, the use of control groups enrolled over the same study period would have limited this bias.

## Conclusions

In this retrospective single center study of patients with ARDS transported to a tertiary referral center, the rate of transport-related complications was slightly above 30% in patients transport in PP, similar to that observed in patients transported in SP without VV-ECMO. In the specific context of our center, our results suggest that transport in the prone position of patients with severe ARDS was safe in selected patients and with the help of specialized transport teams. Finally, VV-ECMO was associated with a non-significantly lower rate of complications in this cohort, which suggest that extracorporeal support could be the best option to secure inter-hospital transportation of severe ARDS patients.

### Supplementary Information

**Additional file 1:** **Supplemental Methods 1.** Initial ventilatory optimization. **Supplemental Methods 2.** VV-ECMO implantation and mechanical ventilation settings during VV-ECMO. **Supplemental Methods 3.** Post-transport patient management in the referral ICU. **Supplemental Methods 4.** Data collection. **Supplemental Table 1.** Missing values. **Supplemental Table 2.** ARDS risk factors. **Supplemental Table 3.** Theoretical indications and potential contra-indications for VV-ECMO in patients transported in the prone or supine position without VV-ECMO. **Supplemental Table 4.** Comparison of patients with or without COVID-19 ARDS. **Supplemental figure 1.** Mechanical ventilation optimization procedure. **Supplemental Figure 2.** Study flow chart. **Supplemental Figure 3.** Transport complications in each study group based on the distance of transport to the referral center. **Supplemental Figure 4.** Frequency of use of prone positioning over the first 7 days after transport in each study group.

## Data Availability

The datasets used and/or analyzed during the current study are available from the corresponding author on reasonable request.
