# Supplementary material for: Safety of inter-facility transport strategies for patients referred for severe acute respiratory distress syndrome
Source: BMC Emerg Med. 2023 Nov 4;23:129. doi: 10.1186/s12873-023-00901-y (PMC10625194; doi:10.1186/s12873-023-00901-y)
Supplement: Supplementary file 1 — Additional file 1: Supplemental Methods 1. Initial ventilatory optimization. Supplemental Methods 2. VV-ECMO implantation and mechanical ventilation settings during VV-ECMO. Supplemental Methods 3. Post-transport patient management in the referral ICU. Supplemental Methods 4. Data collection. Supplemental Table 1. Missing values. Supplemental Table 2. ARDS risk factors. Supplemental Table 3. Theoretical indications and potential contra-indications for VV-ECMO in patients transported in the prone or supine position without VV-ECMO. Supplemental Table 4. Comparison of patients with or without COVID-19 ARDS. Supplemental figure 1. Mechanical ventilation optimization procedure. Supplemental Figure 2. Study flow chart. Supplemental Figure 3. Transport complications in each study group based on the distance of transport to the referral center. Supplemental Figure 4. Frequency of use of prone positioning over the first 7 days after transport in each study group. [file 12873_2023_901_MOESM1_ESM.pdf]

Safety of inter-facility transport strategies for patients referred for severe acute respiratory distress syndrome

Authors: Malik HAOUTAR[1], David PINERO[2,3], Hodane YONIS[1], Eric CESAREO[2,3], Mehdi MEZIDI[1], Olivier PEGUET[2,3], Karim TAZAROURTE[2,3,4], Matteo POZZI[4,5], Pierre-Yves DUBIEN[2,3], Jean-Christophe RICHARD[1,6], Laurent BITKER[1,6]

APPENDIX

Supplemental Methods 1. Initial ventilatory optimization

In volume-controlled mode, tidal volume (V_T) was set at 6 ml.kg^{-1} of predicted body weight (PBW), or less in case of an end-inspiratory airway pressure > 30 cmH_2O at minimal PEEP level [1]. The inspired O_2 fraction (F_iO_2) was set to target an arterial O_2 partial pressure (P_aO_2) between 55 and 80 mmHg, or a S_pO_2 between 88 and 92%. The respiratory rate was set to maintain the intrinsic PEEP < 1 cmH_2O and the pH > 7.20. The I:E ratio was set between 1:2 and 1:3, with a constant inspiratory flow. PEEP was set following a PEEP-F_iO_2 concordance table, and was adjusted based on its effects on oxygenation and systemic hemodynamics [2]. Inhaled nitric oxide (NO) was introduced in case of a P_aO_2 < 55 mmHg or a S_pO_2 < 88% despite initial ventilatory management, at a dose of 10 ppm. Effective neuromuscular blockade was systematically administered by mean of a continuous infusion of cisatracrium or atracrium.

Supplemental Methods 2. VV-ECMO implantation and mechanical ventilation settings during VV-ECMO

VV-ECMO implantation was performed by percutaneous approach with ultrasound guiding. We used Rotaflow (Getinge, Rastatt, Germany), Xenios (Xenios, Fresenius Medical Care, Heilbronn, Germany) and Cardiohelp (Maquet, Rastatt, Germany) VV-ECMO consoles, with Alone (Euroset, Medolla, Italy), PLS (Maquet, Rastatt, Germany), Novalung (Xenios, Fresenius Medical Care, Heilbronn, Germany), and HLS (Maquet, Rastatt, Germany) membrane oxygenators. Anticoagulation under VV-ECMO was performed with unfractionated heparin.

Patients on VV-ECMO were ventilated using quasi-apneic ventilation (volume-controlled mode, tidal volume 1 ml.kg^{-1} PBW, PEEP to maintain end-inspiratory airway pressure between 20 and 25 cmH2O, respiratory rate 5 bpm, I:E ratio of 1:2) or ultra-protective ventilation (pressure controlled mode, PEEP 14 cmH2O, inspiratory pressure 22 cmH2O, respiratory rate 5 bpm, I:E ratio of 1:2). VV-ECMO was set with the following parameters: equal F_iO_2 and F_mO_2 (sweep gas O_2 fraction) set at 1, pump flow corresponding to at least 60% of the patient's measured cardiac output and adjusted to keep the S_pO_2 > 88% and P_aO_2 > 55 mmHg, sweep gas flow equal to the pump flow and adjusted to maintain the arterial CO_2 partial pressure (P_aCO_2) below 45 mmHg.

Supplemental Methods 3. Post-transport patient management in the referral ICU

Patients without VV-ECMO

After transport to the ARDS referral center, patients without VV-ECMO were managed following the most recent French and international ARDS recommendations, including protective ventilation, PEEP titration and weaning, neuromuscular blocking agents administration and PP [3,4]. Patients were placed under VV-ECMO if eligible using the same criteria as given above. Mechanical ventilation weaning was assessed daily, by mean of a spontaneous breathing trial, after PEEP weaning in conscious patients with a $F_iO_2 \leq 50\%$.

Patients with VV-ECMO

Patients under VV-ECMO were ventilated with quasi-apneic ventilation, using the same strategy as given above. VV-ECMO blood and sweep gas flows were adjusted based on the following parameters: S_pO_2 between 88 and 92%, P_aO_2 between 55 and 80 mmHg, $PaCO_2$ < 45 mmHg, pH > 7.20. VV-ECMO weaning was assessed daily, by turning off the sweep gas flow to 0 $L.min^{-1}$ and resuming conventional protective ventilation settings. If the test was positive (based on a P_aO_2/F_iO_2 ratio > 150 mmHg after 2h, and a driving pressure < 15 cmH_2O at V_T 6 $ml.kg^{-1}$ PBW), VV-ECMO was withdrawn on the same day.

Supplemental Methods 4. Data collection

The pre-transport data were taken from the digital file transmitted by the addressing ICU. The per- and post-transport data were collected from the patient's electronic medical record. We identified demographics and comorbidities at baseline, as well as ARDS risk factors and severity, respiratory mechanics and physiology, ARDS therapeutics, SOFA scores, and hospital and ICU mortalities at baseline and during follow-up. Follow-up ended at patient's date of hospital discharge, ICU discharge or death, whichever occurred last.

ARDS class was defined based on the worst P_aO_2/F_iO_2 ratio measured on transport day prior to transport. Oxygenation response to PP was defined by an increase > 20 mmHg in P_aO_2/F_iO_2 ratio between SP and PP at any time on transport day. Data regarding VV-ECMO settings and management (including post-transport implantation) were collected at the same time points.

In case of a missing P_aO_2, it was estimated from the S_pO_2, using the EPIC-II trial concordance table [5]. Likewise, in case of missing S_pO_2, it was estimated from the P_aO_2. The driving pressure was calculated as the difference between the end-inspiratory airway pressure and the total PEEP if measured; else, the set PEEP was used for its computation.

**SpO_2, %**	**PaO_2, mmHg**
81	44
81	45
82	46
83	47
84	49
85	50
86	52
87	53
88	55
89	57
90	60
91	62
92	65
93	69
94	73
95	79
96	86
97	96
98	112
99	145

SpO_2 denotes transcutaneous oxygen saturation, and PaO_2 oxygen partial pressure in arterial blood.

Supplemental Table 1

Supplemental Table 1. Missing values

Variable	N (%)	Variable	N (%)
Age, years	0 (0%)	Distance, km	0 (0%)
Gender male, n (%)	0 (0%)	Duration, min	39 (29%)
Weight, kg	0 (0%)	Transport supervision	
Height, cm	0 (0%)	ARDS centre expert, n (%)	9 (7%)
BMI, kg.m^{-2}	0 (0%)	Mobile ICU expert, n (%)	3 (2%)
Predicted body weight, kg	0 (0%)	Secondary reinforcement by expert, n (%)	27 (20%)
SAPS II score (addressing ICU)	4 (3%)	Transported with inhaled NO, n (%)	18 (13%)
Charlson score	11 (8%)	Ventilatory settings during transport	
Admission category (addressing ICU)	0 (0%)	SpO_2, %	34 (25%)
ARDS severity	0 (0%)	Estimated PaO_2/FiO_2	34 (25%)
ARDS risk factors	0 (0%)	FiO_2 or FmO_2, %	31 (23%)
Delay between ICU admission and transport day, days	1 (1%)	Tidal volume, ml.kg^{-1} PBW	35 (26%)
Delay between intubation and transport day, days	0 (0%)	Set PEEP, cmH_2O	34 (25%)
Transported at night (18H30-8H30) or end of the week days, n (%)	26 (19%)	Plateau pressure, cmH_2O	95 (71%)
Arterial blood gas and ventilatory settings on transport day		Driving pressure, cmH_2O	95 (71%)
SpO_2, %	6 (4%)	Respiratory interventions during transport	
pH	11 (8%)	FiO_2 change, n (%)	31 (23%)
PaO_2/FiO_2 closest to transport, mmHg	4 (3%)	Tidal volume change, n (%)	31 (23%)
PaO_2/FiO_2 in SP on transport day, mmHg	23 (17%)	PEEP change, n (%)	31 (23%)
PaO_2/FiO_2 in PP on transport day, mmHg	22 (17%)	Inhaled NO dose change, n (%)	31 (23%)
$PaCO_2$, mmHg	11 (8%)	Prone to supine positioning during transport, n (%)	26 (19%)
Tidal volume, ml.kg^{-1} PBW	18 (14%)	Supine to prone positioning during transport, n (%)	26 (19%)
Set PEEP, cmH_2O	15 (11%)	Transport related-complications	26 (19%)
Plateau pressure, cmH_2O	45 (34%)		
Driving pressure, cmH_2O	45 (34%)	Outcomes	
Patient management up to transport day		ICU length of stay, days	6 (4%)
Neuromuscular blockade, n (%)	2 (2%)	Hospital length of stay, days	16 (12%)
Inhaled nitric oxide, n (%)	7 (5%)	ICU death, n (%)	2 (1%)
Prone position, n (%)	2 (2%)	In-hospital death, n (%)	2 (1%)
Number of prone sessions before transport day	15 (11%)	Death at day-60, n (%)	2 (1%)
Severity of disease on transport day		Ventilator-free days at day-60, days	7 (5%)
Vasopressors, n (%)	2 (2%)	Alive and free from mechanical ventilation at day-60, n (%)	4 (3%)
Renal replacement therapy, n (%)	3 (2%)	Post-transport VV-ECMO cannulation, n (%)	53 (38%)
SOFA score on transport day, n (%)	17 (13%)	Time to VV-ECMO decannulation, hours	102 (76%)
Lactate, mmol/L	33 (25%)	Alive and free from VV-ECMO at day-60, days	34 (25%)

Supplemental Table 2

Supplemental Table 2. ARDS risk factors

	Whole population n=134	Prone position n=11	VV-ECMO n=44	Supine position n=79	*p*
ARDS severity					
Severe, n (%)	127 (95%)	11 (100%)	43 (98%)	73 (92%)	0.66
ARDS risk factors					
Viral pneumonia, n (%)	106 (79%)	5 (45%)*,†	39 (89%)	62 (78%)	0.01
COVID-19 pneumonitis, n (%)	92 (69%)	2 (18%)*,†	35 (80%)	55 (70%)	<0.01
Bacterial pneumonitis, n (%)	22 (16%)	7 (64%)*,†	4 (9%)	11 (14%)	<0.01
Aspiration pneumonia, n (%)	9 (7%)	2 (18%)	1 (2%)	6 (8%)	0.13
Non-pulmonary sepsis, n (%)	0 (0%)	0 (0%)	0 (0%)	0 (0%)	-
Pancreatitis, n (%)	2 (1%)	0 (0%)	1 (2%)	1 (1%)	0.99
Trauma, n (%)	3 (2%)	0 (0%)	0 (0%)	3 (4%)	0.65
Transfusion, n (%)	0 (0%)	0 (0%)	0 (0%)	0 (0%)	-
Other risk factor, n (%)	6 (4%)	1 (9%)	2 (5%)	3 (4%)	0.52
Unidentified risk factor, n (%)	2 (1%)	0 (0%)	0 (0%)	2 (3%)	0.57

Data is shown as count (percentage). Missing values were not imputed. Percentage are reported to the whole number of observations of the column, including missing values. *p* values examines the difference between groups, using a Fisher test. A post-hoc analysis with pairwise comparison (using a logistic regression model) was performed if the p value of the Fisher test was < 0.20. *: p<0.05 in post hoc analysis between the PP group and the VV-ECMO group; †:p<0.05 in post hoc analysis between the PP group and the SP group. ARDS: acute respiratory distress syndrome; VV-ECMO: veno-venous extracorporeal membrane oxygenation; PP: prone position; SP: supine position

Supplemental Table 3

Supplemental Table 3. Theoretical indications and potential contra-indications for VV-ECMO in patients transported in the prone or supine position without VV-ECMO

	Prone position<br>n=11	Supine position<br>n=79	*p*
Theoretical VV-ECMO indications			
$P_aO_2/F_iO_2 < 50$ mmHg for > 3 hours*	0 (0%)	8 (10%)	0.59
$P_aO_2/F_iO_2 < 80$ mmHg for > 6 hours**	6 (55%)	51 (65%)	0.71
Arterial pH < 7.15 for > 6 hours with a $P_aCO_2 > 60$ mm Hg despite a RR 35 min^{-1} and a plateau pressure ≥ 30 cm H_2O	0 (0%)	0 (0%)	-
At least one indication for VV-ECMO	6 (55%)	51 (65%)	0.52
Theoretical VV-ECMO contra-indications			
Age > 75 years	0 (0%)	1 (1%)	0.99
Left ventricular ejection fraction < 25%	0 (0%)	0 (0%)	-
Chronic disease with < 5-year survival	1 (9%)	4 (5%)	0.49
Chronic respiratory disease	0 (0%)	21 (27%)	0.06
Duration of mechanical ventilation > 7 days	2 (18%)	13 (16%)	0.99
Uncontrolled shock***	1 (9%)	5 (6%)	0.99
SOFA score > 18	1 (9%)	2 (3%)	0.30
Therapeutic limitation decision regarding ECMO	0 (0%)	0 (0%)	-
At least one contra-indication to VV-ECMO	4 (36%)	40 (51%)	0.52
Patients with a VV-ECMO indication and no contra-indication	5 (45%)	26 (33%)	0.50
Number of eligible patients cannulated after transport†	3 (60%)	11 (42%)	0.37

Count and percentage are reported to the whole number of observations of the column, including missing values. All variables were defined in the case report form, except for those identified with one or more *.

*: defined a posteriori using the worst P_aO_2/F_iO_2 value measured on transport day, prior to transport; **: defined a posteriori using the combination of the worst P_aO_2/F_iO_2 value measured on transport day with the P_aO_2/F_iO_2 value measured closest to transport, both being prior to transport; ***: defined a posteriori, using the combination of vasopressor administration and an arterial lactate concentration > 4 $mmol.L^{-1}$; †:denominator is the number of patients with a theoretical ECMO indication and o contra-indications. *p*-values examines the difference between groups using a Fisher test.

F_iO_2: inspired fraction in O_2; P_aO_2: arterial partial pressure in O_2; SOFA: sepsis-related organ failure assessment; VV-ECMO: veno-venous extracorporeal membrane oxygenation

Supplemental Table 4

Supplemental Table 4. Comparison of patients with or without COVID-19 ARDS

	Non-COVID-19 ARDS n=42	COVID-19 ARDS n=92	*p*
**Demographics**			
Age, years	51 [35–65]	56 [47–63]	0.26
Gender male, n (%)	26 (62%)	67 (73%)	0.23
BMI, $kg.m^{-2}$	28 [24–33]	32 [28–36]	<0.01
**Arterial blood gas on transport day**			
Severe ARDS, n (%)	39 (93%)	89 (97%)	0.38
P_aO_2/F_iO_2 closest to transport, mmHg	69 [57–74]	72 [60–85]	0.12
Lowest P_aO_2/F_iO_2 on transport day, mmHg	66 [55–70]	68 [58–78]	0.14
**Ventilatory settings on transport day**			
Tidal volume, $ml.kg^{-1}$ PBW	6.2 [5.9–6.7]	5.8 [4.8–6.3]	0.01
Set PEEP, cmH_2O	14 [10–15]	12 [9–14]	0.21
Plateau pressure, cmH_2O	30 [26–32]	28 [24–30]	0.18
**Patient management on transport day**			
Neuromuscular blockade, n (%)	42 (100%)	92 (100%)	-
Inhaled nitric oxyde, n (%)	28 (67%)	59 (64%)	0.84
Prone position, n (%)	35 (83%)	87 (95%)	0.07
Number of prone sessions before transport day	1 [1–3]	2 [1–3]	0.01
**Severity of disease on transport day**			
Vasopressors, n (%)	32 (76%)	37 (40%)	<0.01
Renal replacement therapy, n (%)	6 (14%)	2 (2%)	0.01
SOFA score on transport day, n (%)	12 [10–14]	9 [8–12]	<0.01
Arterial lactate, $mmol.L^{-1}$	1.8 [1.4–3.3]	1.6 [1.3–2]	0.13
**Transport description**			
Delay between intubation and transport day, days	2 [0–5]	2 [1–5]	0.64
Distance, km	10 [10–89]	44 [7–89]	0.87
Duration, min	40 [30–62]	50 [30–61]	0.38
**Respiratory parameters and ventilation settings during transport**			

Estimated P_aO_2/F_iO_2	76 [60–86]	69 [56–86]	0.37
F_iO_2 or F_mO_2, %	100 [89–100]	100 [88–100]	0.84
Tidal volume, ml.kg^{-1} PBW	5.5 [4.2–6.4]	5.3 [4.1–6]	0.23
Set PEEP, cmH_2O	14 [10–16]	13 [10–15]	0.46
Plateau pressure, cmH_2O	28 [26–34]	26 [20–29]	0.05
**Transport modality**			<0.01
Transported in PP	9 (21%)	2 (2%)	
Transported with VV-ECMO in SP	9 (21%)	35 (38%)	
Transported in SP without VV-ECMO	24 (58%)	55 (60%)	
**Complications during transport**			
Any complications during transport, n (%)	19 (45%)	21 (23%)	0.01
Hypoxemia episode, n (%)	7 (17%)	15 (16%)	0.81
Hypotensive episode, n (%)	10 (24%)	3 (3%)	<0.01
Lowest recorded MAP, mmHg	72 [59–84]	76 [69–87]	0.27
Cardiac arrest during transport, n (%)	0 (0%)	1 (1%)	>0.99
Cardiac arrest on calendar day of transport (after transport), n (%)	1 (2%)	2 (2%)	>0.99
Death on calendar day of transport (after transport), n (%)	3 (7%)	2 (2%)	0.11
Death on calendar day of transport related to transport complications, n (%)	0 (0%)	1 (1%)	>0.99
**Clinical ouctomes**			
ICU length of stay, days	12 [7–25]	28 [14–42]	<0.01
ICU death, n (%)	21 (50%)	58 (63%)	0.13
Death at day-60, n (%)	20 (48%)	57 (62%)	0.09
Ventilator-free days at day-60, days	0 [0–52]	0 [0–11]	0.01
Post-transport ECMO cannulation, n (%)	13 (31%)	25 (27%)	0.21
Number alive and free from ECMO at day-60, days	6 (14%)	16 (17%)	>0.99

Data is shown as median [interquartile range] or count (percentage). Missing values were not imputed. Percentage are reported to the whole number of observations of the column, including missing values. *p*-values examine the difference between groups, using the Wilcoxon-Mann-Whitney test for continuous variables, and the Fisher exact test for categorial variables. BMI: body mass index; F_iO_2: inspired fraction in O_2; F_mO_2: sweep gas O_2 fraction; ICU: intensive care unit; MAP: mean arterial pressure; P_aO_2: arterial partial pressure in O_2; PEEP: positive end-expiratory pressure; PP: prone position; SOFA: sepsis-related organ failure assessment; SP: supine position; VV-ECMO: veno-venous extracorporeal membrane oxygenation.

Supplemental Figure 1

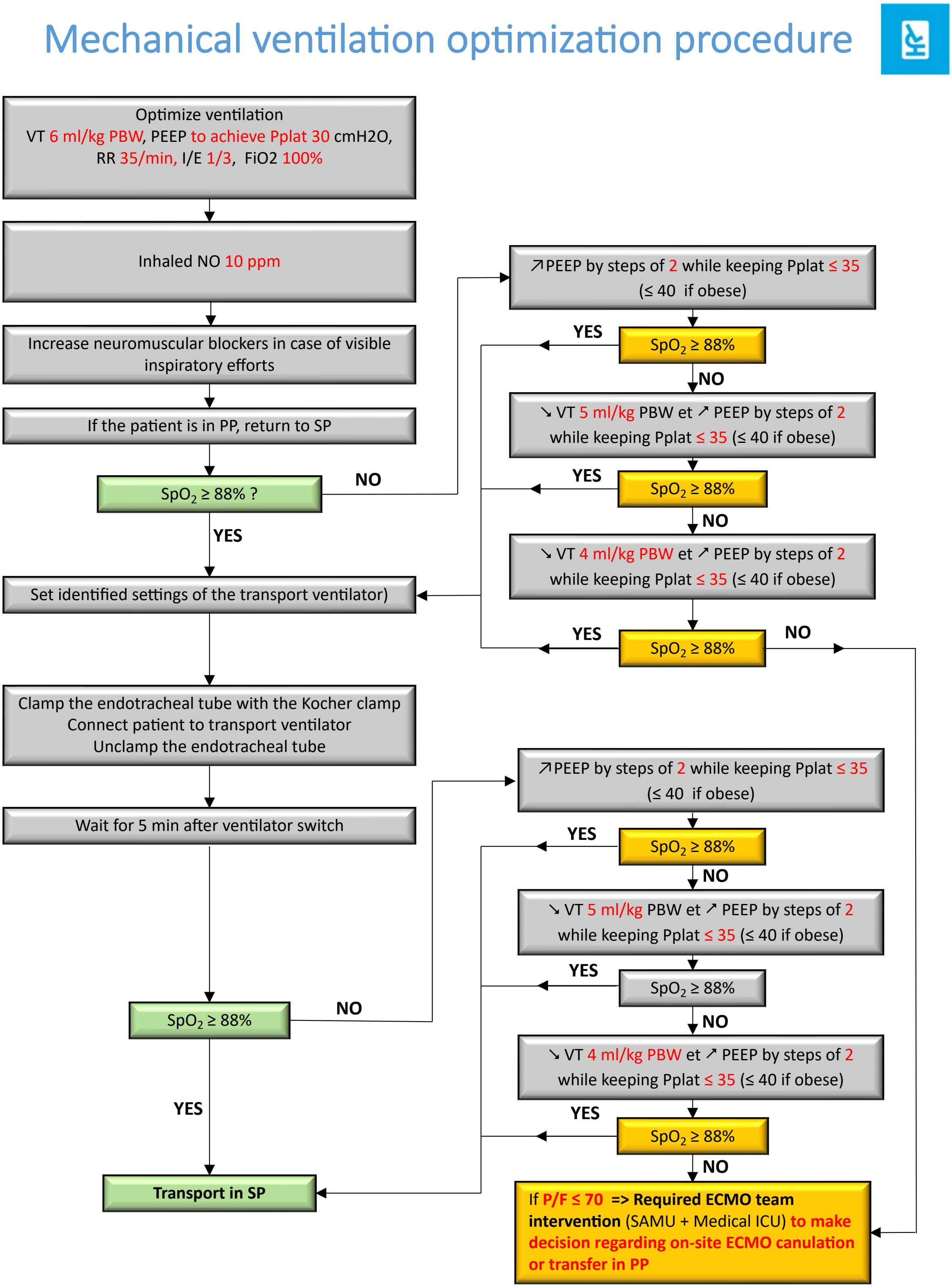

Supplemental figure 1. Mechanical ventilation optimization procedure

The procedure was applied to all patients referred to the Croix Rousse hospital medical ICU, prior to transfer. The aim of the procedure was to maximize lung recruitability while controlling for excessive ventilatory stress, using a stepwise protocol which included lowering tidal volumes and increasing PEEP levels. Transport was deemed safe if a SpO2 ≥ 88% was achieved in SP. In case of refractory hypoxemia despite these adjustments, the mixed VV-ECMO team intervened and decided for on-site cannulation or transport in PP. VV-ECMO: veno-venous extracorporeal membrane oxygenation; FiO2: inspired O_2 fraction; ICU: intensive care unit; NO: nitric oxide; PBW: predicted body weight; PEEP: positive end-expiratory pressure; PP: prone position; P_{PLAT}: plateau pressure; RR: respiratory rate; SAMU: service d'aide médicale d'urgence (emergency medical service); SP: supine position; SpO2: percutaneous O_2 saturation; VT: tidal volume

Supplemental Figure 2

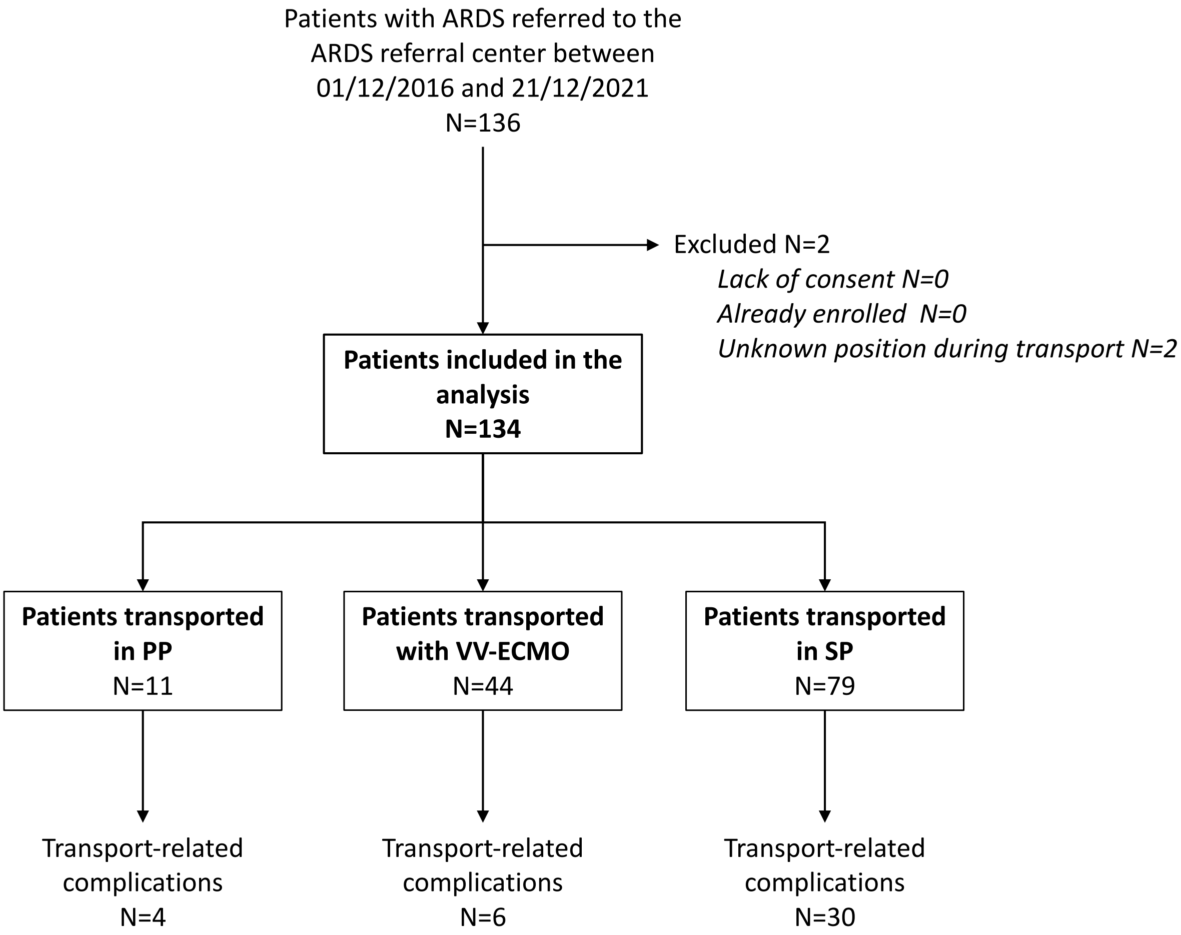

Supplemental Figure 2. Study flow chart

PP: prone position; SP: supine position; VV-ECMO: veno-venous extracorporeal membrane oxygenation

Supplemental Figure 3

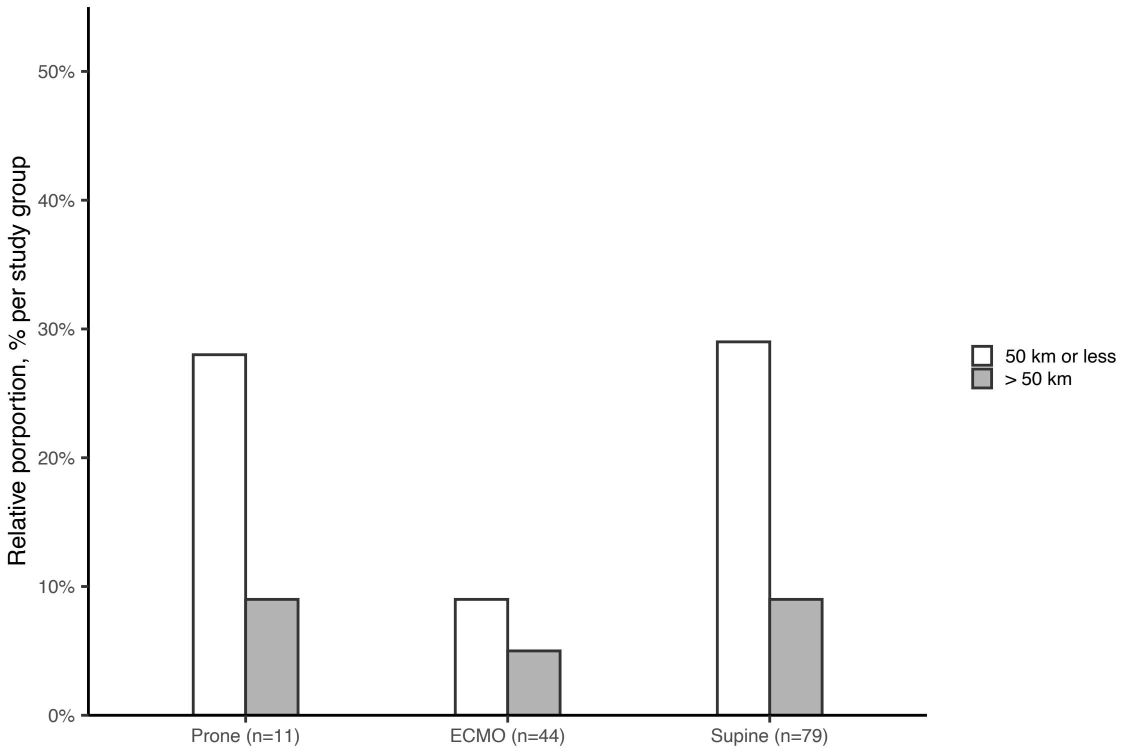

Supplemental Figure 3. Transport complications in each study group based on the distance of transport to the referral center.

The figure shows the relative proportions in each study group of per-transport complications based on the distance in km between the addressing center and the referral center. The distance was dichotomized using an empirical value of 50 km (close to the median value of distance in each study group) for simplification. No significant differences in transport complications incidence was observed between transport distance nor between study groups (no significant interaction between study group and distance). ECMO: extracorporeal membrane oxygenation.

Supplemental Figure 4

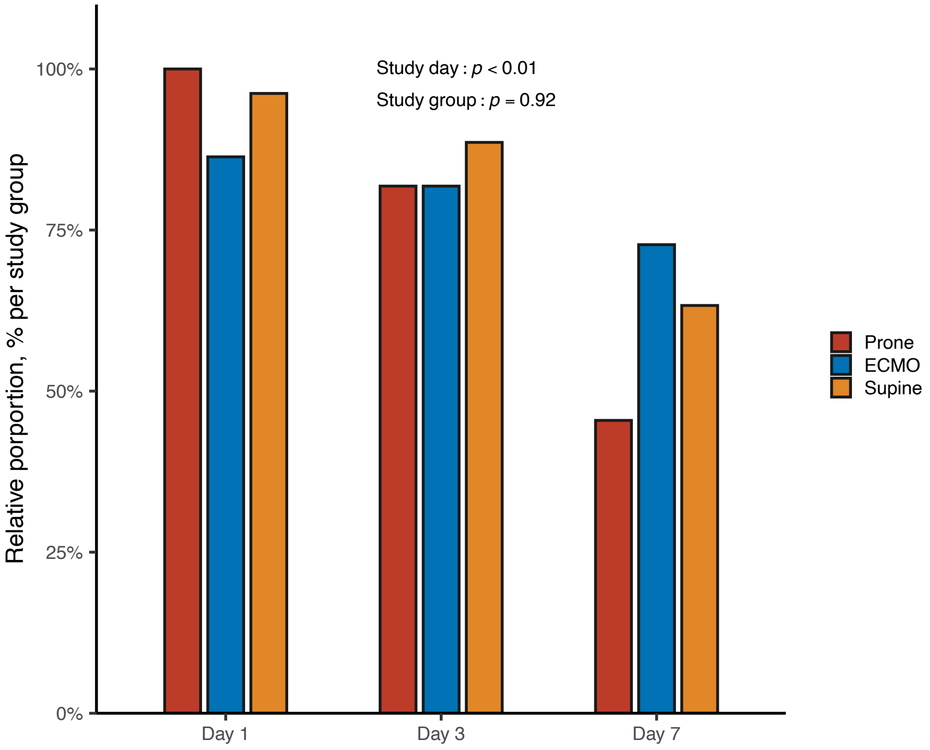

Supplemental Figure 4. Frequency of use of prone positioning over the first 7 days after transport in each study group.

The figure shows the relative proportion of prone positioning, relative to the study group size, in the 3 study groups (prone in red, VV-ECMO in blue, and supine in yellow), on day 1, day 3 and day 7 after transport day. When exploring the association between PP frequency of use over time and study group, no significant interaction between study group and study time was observed. The *p* values hence examine the independent effect of study time and study group on PP frequency. VV-ECMO: veno-venous extracorporeal membrane oxygenation.

References

1. The Acute Respiratory Distress Syndrome Network: Ventilation with lower tidal volumes as compared with traditional tidal volumes for acute lung injury and the acute respiratory distress syndrome. *N Engl J Med* 342 (18): 1301-8, 2000 doi: 10.1056/NEJM200005043421801.

2. Brower RG, Lanken PN, MacIntyre N*, et al*: Higher versus lower positive end-expiratory pressures in patients with the acute respiratory distress syndrome. *N Engl J Med* 351 (4): 327-36, 2004 doi: 10.1056/NEJMoa032193.

3. Papazian L, Aubron C, Brochard L*, et al*: Formal guidelines: management of acute respiratory distress syndrome. *Ann Intensive Care* 9 (1): 69, 2019 doi: 10.1186/s13613-019-0540-9.

4. Fan E, Del Sorbo L, Goligher EC*, et al*: An Official American Thoracic Society/European Society of Intensive Care Medicine/Society of Critical Care Medicine Clinical Practice Guideline: Mechanical Ventilation in Adult Patients with Acute Respiratory Distress Syndrome. *Am J Respir Crit Care Med* 195 (9): 1253-1263, 2017 doi: 10.1164/rccm.201703-0548ST.

5. Vincent JL, Rello J, Marshall J*, et al*: International study of the prevalence and outcomes of infection in intensive care units. *JAMA* 302 (21): 2323-9, 2009 doi: 10.1001/jama.2009.1754.
